# Combined lifestyle factors on mortality among the elder population: evidence from a Chinese cohort study

**DOI:** 10.1186/s12877-022-03017-3

**Published:** 2022-06-01

**Authors:** Changqing Sun, Huimin Liu, Fei Xu, Ying Qin, Panpan Wang, Qianyu Zhou, Dandan Liu, Shanqun Jia, Qiang Zhang

**Affiliations:** 1 School of Public Health, Zhengzhou University, Zhengzhou, China; 2 School of Nursing and Health, Zhengzhou University, Zhengzhou, China

**Keywords:** Healthy lifestyle, Mortality, Cohort study, The elder population

## Abstract

**Background:**

Numerous studies have suggested that lifestyle-related factors are associated with mortality, however limited evidence is available for the Chinese elder population.

**Methods:**

The data of this study was obtained from the Chinese Longitudinal Health Survey (CLHLS) during 2008 − 2018, lifestyle-related factors including body mass index (BMI), smoking, drinking, consumption of vegetables and fruits, physical activity and sleep duration were included as dependent variables in the analysis. A lifestyle risk score was created using six unhealthy behaviors: smoking, drinking, unhealthy weight, physical inactivity, not eat vegetables or fruits and short or prolonged sleep. The Kaplan–Meier curves were used to illustrate the cumulative effect of lifestyle factors on mortality and cox regression models were conducted to estimate the combined effects of lifestyle-related factors on total mortality.

**Results:**

The results illustrated that low BMI, smoking, no fruit eating, and no physical inactivity were risk factors for total mortality. KM curves showed significant cumulative effect of unhealthy lifestyle factors on mortality. Compared with participants without any unhealthy factors, the hazard ratio (*HR*) for participants with six unhealthy factors was 1.335 (1.015,1.757) for all-cause mortality.

**Conclusions:**

This study demonstrated poor adherence to a healthy lifestyle may increase all-cause mortality and specific combinations of lifestyle related factors have different effects on mortality among Chinese elderly population.

**Supplementary Information:**

The online version contains supplementary material available at 10.1186/s12877-022-03017-3.

## Introduction

The risk of developing a major non-communicable disease, the leading cause of death in the world, is decisively affected by lifestyle factors. Lifestyle behaviors such as drinking, smoking, diet, and physical activity lie at the root of many chronic diseases and even mortality [1–4]. Alcohol consumption has been linked to a variety of illnesses including liver cancer, and even light drinkers have a higher risk of death [5]. Cigarette smoking is the leading cause of mortality as a risk factor for lung cancer, coronary heart disease, stroke, chronic obstructive and other diseases [6]. Unhealthy diet such as daily fruit and vegetable intake were inversely associated with all-cause mortality. Other studies with established evidence suggest that lifestyle factors are jointly associated with mortality [7–9]. In a 10-year follow-up cohort study of European individuals, it was estimated that 60% of deaths could be attributed to poor adherence to healthy lifestyle factors including non-smoking, healthy diet, regular physical activity, and moderate drinking [10]. However, the sample size of these studies is relatively small or did not take into account of the emerging lifestyle factors such as short or prolonged sleep [11, 12]. In addition, evidence from prospective studies of Asian countries (especially in China) is limited [13]. Given the difference in lifestyle between Asians and western countries, it is necessary and meaningful to study the relationship between lifestyle and disease or death in Asian population. To understand the effects of these risk factors on disease burden and to provide basic information for the formulation of public health policies and resource allocation, we intended to examine the relationship between six lifestyle behaviors: smoking, unhealthy diet, physical inactivity, body mass index (BMI) abnormal, alcohol consumption and sleep too long or short––and all-cause mortality in Chinese elderly people.

## Data sources and methods

### Data sources

The data of this study was obtained from the Chinese Longitudinal Health Survey (CLHLS) from 2008 to 2018. CLHLS is a national prospective cohort study, which randomly selected 22 counties and cities in China to explore the influencing factors of healthy aging. The study was followed up four times (2008, 2011, 2014, and 2018) during ten years. A multistage cluster sampling approach was used in this prospective, longitudinal, community-based study. After obtaining informed consent, all participants were investigated for demographic characteristics, medical history, lifestyle, and health behaviors, and physical examinations with standardized questionnaire and relevant instruments. Out of 16,954 total respondents who were interviewed in 2008, 2,894 respondents were lost to follow-up in the 2011 survey, 591 respondents were lost to follow-up in the 2014 survey, and 1,259 respondents were lost to follow-up in the 2018 survey. The study was approved by research ethics committees of Peking University (IRB00001052-13,074) and was conducted in accordance with the principles of the Declaration of Helsinki. All the participants signed a consent form.

### Measurements

#### Mortality

Mortality status was determined in the follow-up survey in 2018, we assessed whether the subjects died or not, recorded the year of death, whether the subjects complete the study or were lost to follow-up. Death information was confirmed by a close family member or village doctor. Survival time was calculated from death and baseline, which is the number of years from baseline to the year of death.

#### Lifestyle factors

Lifestyle variables included smoking (never, former, or current), drinking (never, former, or current drinking), physical activity (never, former or current), BMI, fruit consumption, vegetable consumption and sleep duration. Participants were classified into underweight (< 18.5 kg/m^2^), normal weight (18.5–24.9 kg/m^2^), overweight (25.0–29.9 kg/m^2^), and obese (≥ 30.0 kg/m^2^) according to WHO criteria [14]. Participants reported on a range of lifestyle risk behaviors in the questionnaire. Smoking and drinking status were derived from questions: “Are you a smoker/drinker at present?” “Have you smoked/drank in the past?” Fruit and vegetable consumption were measured by frequency and divided into binary categories including eat or not according to the questions of “do you eat fresh fruit?” or “ do you eat fresh vegetables?”. Sleeping time < 7 h or > 9 h per day was defined as poor sleep pattern according to the question of how many hours do you sleep every day. Those lifestyle factors were used to generate a lifestyle score. For each of the six selected lifestyle risk factors, participants received a score of 1 if they practiced the unhealthy behavior, otherwise received a score of 0 [15]. A total lifestyle risk score ranged from 0 to 6, higher scores indicate an unhealthier lifestyle.

#### Covariates

Multiple variables were included in the analysis as covariates. Educational level was divided into three categories (illiteracy, primary school and below, junior high school and above). Marital status (married and living with a patterner, separated, divorced, widowed or single) was divided into 2 categories (have a spouse or not). Self-assessment of health status was classified into poor, general and rich. ADL was estimated using a 0–6 point Katz score scale [16]. Physical disability was defined as a need for assistance or a difficulty in one or more of the six activities listed above.

#### Statistical analyses

Chi-square analysis was first used to examine the gender differences in baseline characteristics. The Kaplan–Meier curves were plotted to illustrate the cumulative effect of lifestyle factors on mortality. In this study, a series of Cox proportional hazard models were used to assess the relationship between lifestyle factors and all-cause mortality, adjusting for multiple covariates. The first model assessed the relationship between lifestyle factors and mortality. Then socio-demographic variables (age, gender, residence, married status and co-residence of the interviewee) were included as covariates in the second model. The third model further included health status including self-report health, ADL and chronic diseases. The effects of responses who died in the first 2 years of follow-up were tested in sensitivity analyses. To examine specific patterns of lifestyle risk behaviors, we generated 64 variables representing all possible combinations of six unhealthy lifestyle behaviors. *P* value < 0.05 was considered to be statistically significant. All the analysis was performed in SPSS 21.0.

## Results

After deleting the miss sample size of personal characteristics and related lifestyle factors, this study sample across follow-up consists of information of 8915 deaths and 2309 alive. Among the sample, 6432 of participants were female, 7294 (64.99%) reported illiteracy, 7762 (68.90%) reported they did not have a spouse, 7724 (68.82%) reported general economic status was 7385 (65.80%) lived in rural, 4918 (43.82%) reported good health status, 8837 (78.73%) reported the ADL was normal and 4939 (44.00%) reported did not have any chronic diseases. Table 1 presented the patient characteristics of the mortality groups (alive vs. dead) in the categories of sociodemographic.

**Table 1:** Baseline characteristics among the elder in China

Characteristic	N	%	Alive	Dead	/^2^(*P*)
Age					(< 0.001)
65 ~	1865	16.62	1250(67.02)	615(32.98)	
75 ~	2312	20.60	751(32.48)	1561(67.52)	
85 ~	3837	34.19	259(6.75)	3578(93.25)	
95 ~	3210	28.60	49(1.53)	3161(98.47)	
Gender					13.9759(< 0.001)
Male	4792	42.69	1065(22.22)	3727(77.78)	
Female	6432	57.31	1244(19.34)	5188(80.66)	
Education level					346.3358(< 0.001)
Illiteracy	7294	64.99	1127(15.45)	6167(84.55)	
Primary school and below	3016	26.87	870(28.85)	2146(71.15)	
Junior high school and above	914	8.14	312(34.14)	602(65.86)	
Marital status					(< 0.001)
Have a spouse	3492	31.11	1405(40.23)	2087(59.77)	
No spouse	7732	68.89	904(11.69)	6828(88.31)	
Self-assessment of economic status					14.4912(< 0.001)
Rich	1426	12.70	291(20.41)	1135(79.59)	
General	7724	68.82	1653(21.40)	6071(78.60)	
Poor	2074	18.48	365(17.60)	1709(82.40)	
Living in					2.8682(0.238)
City	1558	13.88	296(19.00)	1262(81.00)	
Town	2281	20.32	469(20.56)	1812(79.44)	
Rural	7385	65.80	1544(20.91)	5841(79.09)	
Self-reported health					255.8146(< 0.001)
Good	4918	43.82	1276(25.95)	3642(74.05)	
General	3295	29.36	701(21.27)	2594(78.73)	
Poor	3011	26.83	332(11.03)	2679(88.97)	
ADL					
Limited	2387	21.27	46(1.93)	2341(98.07)	645.0073(< 0.001)
Normal	8837	78.73	2263(25.61)	6574(74.39)	
Chronic disease					2.1626(0.339)
No	4939	44.00	986(19.96)	3953(80.04)	
One of chronic disease	3565	31.76	744(20.87)	2821(79.13)	
Two and above	2720	24.23	579(21.29)	2141(78.71)	
Smoking					40.6484(< 0.001)
Never	7434	66.23	1496(20.12)	5938(79.88)	
Former	1802	16.05	310(17.20)	1492(82.80)	
Current	1988	17.71	503(25.30)	1485(74.70)	
Drinking					28.8123(< 0.001)
Never	7651	68.17	1524(19.92)	6127(80.08)	
Former	1570	13.99	288(18.34)	1282(81.66)	
Current	2003	17.85	497(24.81)	1506(75.19)	
Physical activity					162.7729(< 0.001)
Physical inactivity	6957	61.98	1302(18.71)	5655(81.29)	
Former	1413	12.59	195(13.80)	1218(86.20)	
Current	2854	25.43	812(28.45)	2042(71.55)	
BMI					260.7597(< 0.001)
Underweight (< 18.5 kg/m^2^)	3881	34.58	511(13.17)	3370(86.83)	
Normal weight (18.5–24.9 kg/m^2^)	6424	57.23	1483(23.09)	4941(76.91)	
Overweight (25.0–29.9 kg/m^2^)	785	6.99	270(34.39)	515(65.61)	
Obese (≥ 30.0 kg/m^2^)	134	1.19	45(33.58)	89(66.42)	
Vegetable consumption					32.1270(< 0.001)
Yes	10,907	97.18	2284(20.94)	8623(79.06)	
Rarely or never	317	2.82	25(7.89)	292(92.11)	
Fruit consumption					67.3568(< 0.001)
Yes	8259	73.58	1854(22.45)	6405(77.55)	
Rarely or never	2965	26.42	455(15.35)	2510(84.65)	
Sleep duration					108.9058(< 0.001)
7–9 h	4930	43.92	1236(25.07)	3694(74.93)	
< 7/ > 9 h	6294	56.08	1073(17.05)	5221(82.95)	

**Fig. 1:**
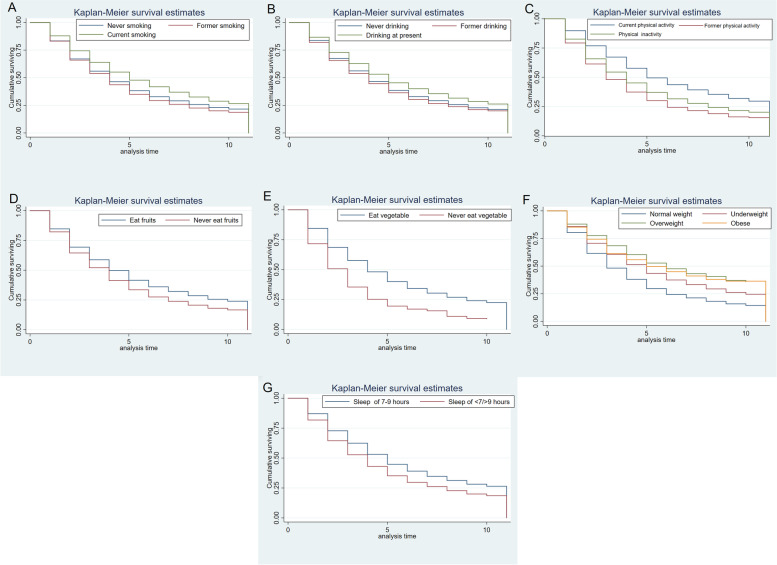
Associations of lifestyle risk with all-cause mortality in the CLHLS (**A**: Smoking; **B**: Drinking; **C**: Physical activity; **D**: BMI; **E**: Vegetable consumption; **F**: Fruit consumption; **G**: Sleep duration)

An association was observed between lifestyle-related factors and the risk of total mortality. Kaplan–Meier curves demonstrated that participants who were current smoking, having physical activity, obesity, eating fruits, eating vegetables and sleeping of 7–9 h tended to live longer (Fig. 1).

Our model did not identify any significant association between drinking and mortality. Surprisingly, the results showed that current smoking was associated with decreased risk of mortality, however, after adjusting for basic demographic information and health status, former smoking and current smoking both showed significant association with increased mortality risk. Compared with participants who were having physical activity at present, the hazard ratios (*HR*) of participants who were physical inactivity or former were 1.110 (1.037, 1.189) and 1.183 (1.105,1.267) (Table 2). The results of age-stratified showed that smoking was associated increased mortality risk in all age groups (Table S2). We separately excluded deaths that occurred in the first 2 years, both of which did not lead to remarkable changes in *HR* estimates (Table S1).

**Table 2:** *HRs* and 95% *CIs* for the mortality risks of healthy lifestyle among the elderly in China

	Model 1	Model 2	Model 3
	*HR(95%CI)*	*HR(95%CI)*	*HR(95%CI)*
Drinking			
Never	1	1	1
Former	1.064(0.993,1.139)	1.058(0.988,1.133)	1.043(0.974,1.118)
Current	0.936(0.879,0.997)	0.953(0.894,1.015)	0.966(0.906,1.029)
Smoking			
Never	1	1	1
Former	1.118(1.048,1.193)	1.201(1.122,1.287)	1.183(1.105,1.267)
Current	0.881(0.827,0.938)	1.092(1.020,1.169)	1.110(1.037,1.189)
Physical activity			
Current	1	1	1
Former	1.456(1.351,1.569)	1.245(1.154,1.342)	1.146(1.062,1.237)
Physical inactivity	1.279(1.213,1.348)	1.172(1.110,1.239)	1.122(1.062,1.186)
Eat fruits			
Yes	1	1	1
Rarely or never	1.152(1.097,1.211)	1.072(1.020,1.127)	1.057(1.006,1.111)
Eat vegetable			
Yes	1	1	1
Rarely or never	1.427(1.257,1.619)	1.209(1.065,1.372)	1.106(0.974,1.255)
BMI			
Normal weight(18.5–24.9 kg/m^2^)	1	1	1
Underweight (< 18.5 kg/m^2^)	1.312(1.253,1.375)	1.068(1.019,1.120)	1.055(1.006,1.105)
Overweight (25.0–29.9 kg/m^2^)	0.773(0.704,0.850)	0.984(0.895,1.081)	0.969(0.882,1.065)
Obese (≥ 30.0 kg/m^2^)	0.803(0.647,0.996)	0.866(0.697,1.075)	0.838(0.675,1.041)
Sleep duration			
7–9 h	1	1	1
< 7/ > 9 h	1.236(1.183,1.291)	1.078(1.032,1.127)	1.032(0.987,1.079)

Model 1. Unadjusted model

Model 2. Model 1 adjusted for age, gender, residential type, marital status, economic situation and education

Model 3. Model 2 adjusted for several chronic diseases, ADL and self-reported health

As the number of high-risk lifestyle behaviors increased, the risk of all-cause mortality increased progressively (Table 3). In comparison with participants without any healthy factors, the *HR* (95%*CI*) of participants who had six unhealthy healthy factors was 1.857 (1.415,2.437) for all-cause mortality, and this association remained significant after adjusting for potential confounders. The association signal remained after excluding the deaths during first 2 years of follow-up (Table S3). And the results of age-stratified analysis showed that having more unhealthy lifestyle risk factors were associated with considerably higher risk of mortality among the 75- and 85- age groups. The HRs of participants who had six unhealthy healthy factors were 1.96 and 1.59 respectively among the 75- and 85- age groups (Table S4).

**Table 3:** *HRs* and 95%*CIs* for the mortality risks of lifestyle among the elderly in China

	Model 1	Model 2	Model 3
	*HR (95%CI)*	*HR (95%CI)*	*HR (95%CI)*
Risk score			
0	1	1	1
1	1.112 (0.974,1.271)	1.087 (0.951,1.243)	1.057 (0.925,1.209)
2	1.349 (1.188,1.532)	1.156 (1.017,1.314)	1.108 (0.974,1.260)
3	1.483 (1.306,1.684)	1.252 (1.100,1.424)	1.175 (1.033,1.337)
4	1.537 (1.346,1.755)	1.328 (1.160,1.519)	1.224 (1.069,1.401)
5	1.753 (1.504,2.044)	1.484 (1.269,1.735)	1.354 (1.158,1.585)
6	1.857 (1.415,2.437)	1.531 (1.164,2.012)	1.339 (1.018,1.762)

Model 1. Unadjusted model

Model 2. Model 1 adjusted for age, gender, residential type, marital status, economic situation and education

Model 3. Model 2 adjusted for several chronic diseases, ADL and self-reported health

We also calculated the *HRs* for the mutually exclusive combinations of high-risk lifestyle factors (Table 4). Among combinations of all risk behaviors, combinations of smoking and unhealthy diet showed the strongest association with all-cause mortality (*HR* = 1.670). For all combinations of two risk factors, smoking plus physical inactivity and smoking plus unhealthy diet showed relatively strong associations with all-cause mortality (*HR* = 1.258; *HR* = 1.342). For three-factor combinations, short or long sleep duration plus physical inactivity plus BMI abnormal (*HR* = 1.174), unhealthy diet plus short or long sleep duration plus physical inactivity (*HR* = 1.287), physical inactivity plus smoking plus BMI abnormal (*HR* = 1.296), smoking plus unhealthy diet plus physical inactivity (*HR* = 1.387), and smoking plus drinking and abnormal BMI (*HR* = 1.308) showed a relatively strong association with all-cause mortality. Among the four-risk combinations, smoking plus unhealthy diet, drinking, short or long sleep duration and BMI abnormal (*HR* = 1.496) had a stronger association with all-cause mortality, whereas the *HR* of drinking plus short or long sleep duration plus physical activity plus BMI abnormal is 1.276. In addition, smoking plus short or long sleep duration plus physical inactivity plus BMI abnormal were associated with all-cause mortality (*HR* = 1.286). Among the five-factor combinations, the combined risks had a stronger association with all-cause mortality except the smoking plus drinking plus short or long sleep duration plus unhealthy diet plus BMI abnormal. The *HR* of all the six lifestyle factors is 1.350 (Table 4).

**Table 4:** *HRs* and 95% *CIs* for the mortality risks of lifestyle among the elderly in China

Smoking	Drinkking	Fruit/vegetable consumption	Sleep duration	Physical activity	BMI	*HR(95%CI)*	*HR(95%CI)*	*HR(95%CI)*
-	-	-	-	-	+	1.136(0.941,1.371)	1.101(0.912,1.330)	1.110(0.919,1.340)
-	-	-	-	+	-	1.050(0.903,1.221)	1.072(0.920,1.249)	1.051(0.902,1.224)
-	-	-	-	+	+	1.323(1.133,1.544)	1.116(0.953,1.306)	1.097(0.937,1.284)
-	-	-	+	-	-	1.271(1.080,1.496)	1.101(0.935,1.296)	1.022(0.868,1.203)
-	-	-	+	-	+	1.329(1.133,1.544)	1.042(0.880,1.235)	0.977(0.824,1.158)
-	-	-	+	+	-	1.478(1.282,1.704)	1.161(1.005,1.340)	1.089(0.942,1.258)
-	-	-	+	+	+	1.831(1.584,2.115)	1.285(1.110,1.489)	1.174(1.013,1.361)
-	-	+	-	-	-	1.229(0.946,1.598)	1.183(0.909,1.538)	1.165(0.895,1.515)
-	-	+	-	-	+	1.388(1.055,1.828)	1.182(0.896,1.558)	1.108(0.840,1.460)
-	-	+	-	+	-	1.549(1.284,1.869)	1.257(1.040,1.519)	1.181(0.976,1.428)
-	-	+	-	+	+	1.757(1.454,2.123)	1.258(1.039,1.523)	1.162(0.959,1.408)
-	-	+	+	-	-	1.552(1.235,1.951)	1.226(0.974,1.542)	1.151(0.914,1.448)
-	-	+	+	-	+	1.535(1.230,1.916)	1.118(0.894,1.397)	1.036(0.828,1.295)
-	-	+	+	+	-	1.909(1.613,2.258)	1.406(1.186,1.667)	1.287(1.085,1.527)
-	-	+	+	+	+	1.897(1.603,2.245)	1.289(1.086,1.530)	1.142(0.961,1.357)
-	+	-	-	-	-	1.048(0.821,1.338)	0.979(0.767,1.251)	0.947(0.741,1.210)
-	+	-	-	-	+	1.275(0.932,1.745)	1.004(0.733,1.374)	1.020(0.745,1.397)
-	+	-	-	+	-	1.035(0.820,1.338)	0.963(0.762,1.217)	0.955(0.756,1.208)
-	+	-	-	+	+	1.308(1.013,1.688)	1.040(0.805,1.344)	1.042(0.806,1.347)
-	+	-	+	-	-	1.460(1.139,1.871)	1.111(0.866,1.424)	1.031(0.804,1.322)
-	+	-	+	-	+	1.759(1.359,2.277)	1.296(1.001,1.678)	1.179(0.911,1.578)
-	+	-	+	+	-	1.395(1.126,1.727)	1.129(0.911,1.400)	1.061(0.856,1.316)
-	+	-	+	+	+	1.803(1.429,2.276)	1.384(1.095,1.749)	1.276(1.010,1.613)
-	+	+	-	-	-	1.321(0.906,1.278)	0.991(0.679,1.447)	0.962(0.659,1.405)
-	+	+	-	-	+	1.329(0.835,2.118)	1.182(0.741,1.885)	1.129(0.708,1.801)
-	+	+	-	+	-	1.254(0.855,1.840)	1.134(0.772,1.666)	1.096(0.746,1.611)
-	+	+	-	+	+	1.703(1.196,2.423)	1.154(0.810,1.645)	1.058(0.742,1.508)
-	+	+	+	-	-	1.482(1.041,2.108)	1.208(0.849,1.720)	1.193(0.838,1.700)
-	+	+	+	-	+	1.840(1.211,2.795)	1.225(0.806,1.861)	1.247(0.821,1.895)
-	+	+	+	+	-	1.642(1.240,2.173)	1.202(0.907,1.593)	1.131(0.853,1.501)
-	+	+	+	+	+	2.515(1.849,3.422)	1.503(1.103,2.048)	1.377(1.010,1.877)
+	-	-	-	-	-	0.933(0.733,1.187)	1.117(0.876,1.425)	1.130(0.887,1.441)
+	-	-	-	-	+	1.333(1.024,1.734)	1.237(0.949,1.613)	1.261(0.967,1.643)
+	-	-	-	+	-	1.155(0.924,1.444)	1.285(1.026,1.610)	1.258(1.004,1.577)
+	-	-	-	+	+	1.450(1.136,1.852)	1.328(1.038,1.698)	1.296(1.013,1.659)
+	-	-	+	-	-	1.487(1.203,1.834)	1.416(1.144,1.752)	1.342(1.084,1.661)
+	-	-	+	-	+	1.468(1.146,1.881)	1.285(1.002,1.648)	1.163(0.907,1.493)
+	-	-	+	+	-	1.274(1.033,1.551)	1.166(0.944,1.441)	1.123(0.909,1.388)
+	-	-	+	+	+	1.770(1.407,2.226)	1.400(1.111,1.763)	1.286(1.021,1.621)
+	-	+	-	-	-	1.493(1.063,2.099)	1.730(1.230,2.433)	1.670(1.187,2.350)
+	-	+	-	-	+	1.550(1.079,2.226)	1.081(0.752,1.553)	1.062(0.739,1.526)
+	-	+	-	+	-	1.758(1.299,2.379)	1.423(1.050,1.930)	1.386(1.022,1.881)
+	-	+	-	+	+	1.456(1.040,2.038)	1.206(0.860,1.692)	1.072(0.764,1.504)
+	-	+	+	-	-	1.179(0.829,1.678)	1.207(0.848,1.719)	1.086(0.762,1.547)
+	-	+	-	+	-	2.086(1.453,2.996)	1.561(1.085,2.244)	1.496(1.040,2.152)
+	-	+	-	-	+	1.621(1.250,2.100)	1.405(1.081,1.826)	1.274(0.980,1.657)
+	-	+	+	+	+	2.093(1.578,2.776)	1.783(1.343,2.369)	1.591(1.197,2.114)
+	+	-	-	-	-	1.019(0.838,1.238)	1.153(0.946,1.404)	1.133(0.930,1.381)
+	+	-	-	-	+	1.212(0.956,1.537)	1.312(1.033,1.667)	1.3081.029,1.662)
+	+	-	-	+	-	1.010(0.830,1.229)	1.238(1.013,1.512)	1.183(0.968,1.445)
+	+	-	-	+	+	1.313(1.038,1.662)	1.369(1.080,1.737)	1.333(1.050,1.692)
+	+	-	+	-	-	1.133(0.938,1.370)	1.235(1.020,1.496)	1.194(0.986,1.447)
+	+	-	+	-	+	1.305(1.041,1.636)	1.343(1.069,1.687)	1.244(0.990,1.564)
+	+	-	+	+	-	1.277(1.066,1.530)	1.389(1.156,1.670)	1.298(1.079,1.560)
+	+	-	+	+	+	1.797(1.462,2.210)	1.488(1.207,1.836)	1.360(1.102,1.678)
+	+	+	-	-	-	1.147(0.865,1.521)	1.199(0.902,1.594)	1.170(0.879,1.555)
+	+	+	-	-	+	1.241(0.803,1.943)	1.070(0.692,1.656)	0.973(0.628,1.506)
+	+	+	-	+	-	1.488(1.140,1.943)	1.487(1.136,1.946)	1.410(1.077,1.846)
+	+	+	-	+	+	1.719(1.245,2.373)	1.502(1.086,2.079)	1.410(1.018,1.950)
+	+	+	+	-	-	1.258(0.955,1.656)	1.174(0.890,1.550)	1.126(0.853,1.486)
+	+	+	+	-	+	1.695(1.224,2.347)	1.454(01.048,2.017)	1.280(0.923,1.776)
+	+	+	+	+	-	1.427(1.137,1.791)	1.350(1.072,1.700)	1.267(1.006,1.596)
+	+	+	+	+	+	1.866(1.422,2.449)	1.533(1.166,2.017)	1.350(1.026,1.777)

***Note:*** “ + ” represents the risk score was 1, “-” represents the risk score was 0

## Discussion

In this prospective cohort study of Chinese elderly individuals, we estimated the risk of all-cause mortality over 10 years of follow-up for six lifestyle factors and 64 possible combinations of lifestyle factor components. Our results showed that as the number of unhealthy lifestyle factors increases, the mortality increases. Compared with participants without any unhealthy factors, the *HR* (95%*CI*) of participants who had six unhealthy healthy factors was 1.339 (1.018,1.762) for all-cause mortality. Our results are consistent with the results of several other studies that also related high-risk lifestyle factors to all-cause mortality [17–19].This indicate that healthy lifestyle behaviors should focus on many factors to prevent disease and mortality.

Our study found that low BMI, smoking, no fruit consumption, and physical inactivity were risk factors for total mortality. In this study, individuals with underweight had a higher risk of total mortality. Mortality was minimized in older individuals at higher BMI, perhaps indicating increased importance of nutritional reserves in older age, but after adjusting the confounders, there is no association between BMI and mortality. Some studies reported a U-shaped or J-shaped association between BMI and all-cause mortality [20–23]. Other studies showed that underweight (BMI < 18.5 kg/m^2^) was significantly associated with risk for all-cause mortality as compared with normal weight (BMI = 18.5–22.9 kg/m^2^), while obesity (BMI ≥ 30.0 kg/m^2^) was not associated with excess mortality risk, which is consistent with our study [24–26]. This finding might suggest that healthy weight recommendations need to account for age and other related factors, but further work is needed to establish whether increased weight is beneficial for older individuals and how it works [27]. The current and former smokers had a higher risk of all-cause mortality than never smokers consistent with an elderly Chinese cohort study [28]. Alcohol consumption was not associated with an increased risk of mortality in this study. The result was consistent with another systematic review which reported the magnitude of risk posed by alcohol use for mortality among older adults remains uncertain [29]. However, a Longitudinal study in Europe found that moderate alcohol consumption (HR, 0.83; 95% CI, 0.71–0.91) was associated with a significantly lower risk of all-cause mortality [10]. Whether protective or adverse effects dominate for drinkers averaging a small volume of drinking is likely to depend on age and cultural, social, environmental, and genetic factors and on whether their drinking includes heavy drinking occasions [30]. The data about type of alcohol consumed and the amount of alcohol consumed is limited and whether type of alcohol consumed and the amount of alcohol consumed have an impact on mortality still needs further study.

It is important to acknowledge that not all risk behaviors contribute to mortality similarly and that their combined effects may not be additive [31]. Some risk behaviors tend to cluster together, and the combined risks may be much higher than the sum of individual risks. The HR of smoking plus drinking and BMI abnormal is 1.308, though alcohol consumption on its own was not significantly associated with higher mortality risk. The combination of two risk factors – smoking and low intake of fruits or vegetables – was higher than the combination of six risk factors. These findings suggest that future epidemiological research and behavioral interventions should take into account the patterns of risk factor co-occurrence and their interactive effects on health outcomes. We need to pay attention to the interaction of physical exercise, smoking and dietary patterns in the elderly.

There are several advantages in this study. First, this is a large-scale longitudinal study performed in Chinese elderly populations, and the participants were randomly recruited from general population, therefore the results may have strong generalization to the national elderly population. In addition, an emerging lifestyle factor sleeping was also included in the current study, and the different combination of lifestyle factors in this study allows us to more accurately examine the effect of different risk factors on mortality. Furthermore, three different models were applied to control the influence of multiple covariates including health and demographic indicators. Finally, as a sensitivity analysis, we excluded deaths in the previous two years and further adjusted for additional covariates. However, our study still has some limitations. First, because of the diet complexity of Chinese residents, we did not use dietary information to evaluate overall diet status, and further study were expected to assess the relationship between healthy diet and mortality. Secondly, the changes in lifestyle factors during the follow-up were not available in this study, which may affect the results. Thirdly, we explored the association between lifestyle factors and mortality, but did not included different sub-types of mortality because of the limit of the database. Moreover, we did not collect more detailed information on smoking and alcohol drinking, which made it difficult to conduct further dose − response analysis.

## Conclusions

In this cohort, participants who were underweight, smoking, drinking, physical inactivity and unhealthy diet were significantly associated with a higher risk of all-cause mortality. As the number of high-risk lifestyle behaviors increases, so does the risk of all-cause mortality. Our study showed a strong association between the combined effects of lifestyle-related factors and the risk of all-cause mortality.

## Supplementary Information

**Additional file 1:** **Table S1. ***HRs* and 95%*CIs* for the mortality risks of healthy lifestyle among the elderly in China. **Table S2. ***HRs*and 95% *CIs* for the mortality risks of healthy lifestyle among the elderly by age in China. **Table S3. ***HRs* and 95%*CIs *for the mortality risks of lifestyle among the elderly in China. **Table S4. ***HRs* and 95%*CIs* for the mortality risks of lifestyle among the elderly by age in China.

## Data Availability

The data that support the findings of this study are available from the Chinese Longitudinal Health Survey, but restrictions apply to the availability of these data, which were used under license for the current study, and so are not publicly available. Data are however available from the authors upon reasonable request.

## Abbreviations

BMI: Body mass index
ADL: Activity of daily living
CLHLS: Chinese longitudinal healthy longevity survey
