# Supplementary material for: Combined lifestyle factors on mortality among the elder population: evidence from a Chinese cohort study
Source: BMC Geriatr. 2022 Jun 1;22:474. doi: 10.1186/s12877-022-03017-3 (PMC9158191; doi:10.1186/s12877-022-03017-3)
Supplement: Supplementary file 1 — Additional file 1: Table S1. HRs and 95%CIs for the mortality risks of healthy lifestyle among the elderly in China. Table S2. HRsand 95% CIs for the mortality risks of healthy lifestyle among the elderly by age in China. Table S3. HRs and 95%CIs for the mortality risks of lifestyle among the elderly in China. Table S4. HRs and 95%CIs for the mortality risks of lifestyle among the elderly by age in China. [file 12877_2022_3017_MOESM1_ESM.doc]

**[Supplementary](C:/Users/minmin/AppData/Local/youdao/dict/Application/8.9.3.0/resultui/html/index.html#/javascript:;) [tables](C:/Users/minmin/AppData/Local/youdao/dict/Application/8.9.3.0/resultui/html/index.html#/javascript:;):**

**Table S1. *HRs* and 95%*CIs* for the mortality risks of healthy lifestyle among the elderly in China**

|  | Model 1 | Model 2 | Model 3 |
| --- | --- | --- | --- |
|  | *HR（95%CI）* | *HR（95%CI）* | *HR（95%CI）* |
| Drinking |  |  |  |
| Never | 1 | 1 | 1 |
| Former | 1.035(0.958,1.119) | 1.025(0.949,1.108) | 1.012(0.937,1.094) |
| Current | 0.926(0.864,0.994) | 0.946(0.881,1.015) | 0.957(0.891,1.027) |
| Smoking |  |  |  |
| Never | 1 | 1 | 1 |
| Former | 1.146(1.065,1.232) | 1.233(1.142,1.332) | 1.217(1.127,1.315) |
| Current | 0.908(0.846,0.973) | 1.125(1.043,1.213) | 1.141(1.059,1.232) |
| Physical activity |  |  |  |
| Current | 1 | 1 | 1 |
| Former | 1.380(1.268,1.501） | 1.191(1.094,1.297) | 1.108(1.017,1.208) |
| Physical inactivity | 1.221(1.152,1.293) | 1.129(1.063,1.200) | 1.093(1.028,1.161) |
| Fruit |  |  |  |
| Yes | 1 | 1 | 1 |
| Rarely or never | 1.174(1.111,1.241) | 1.080(1.021,1.142) | 1.063(1.004,1.124) |
| Vegetable |  |  |  |
| Yes | 1 | 1 | 1 |
| Rarely or never | 1.362(1.170,1.585) | 1.152(0.990,1.341) | 1.067(0.916,1.242) |
| BMI |  |  |  |
| Normal weight(18.5-24.9kg/m²) | 1 | 1 | 1 |
| Underweight (<18.5 kg/m²) | 1.314(1.247,1.384) | 1.056(1.001,1.113) | 1.044(0.990,1.101) |
| Overweight (25.0-29.9 kg/m²) | 0.752(0.678,0.835) | 0.968(0.872,1.075) | 0.954(0.859,1.060) |
| Obese (≥30.0 kg/m²) | 0.755(0.590,0.965) | 0.817(0.639,1.045） | 0.797(0.623,1.019) |
| Sleep duration |  |  |  |
| 7-9 hours | 1 | 1 | 1 |
| <7/>9 hours | 1.210(1.152，1.293） | 1.053(1.002,1.106） | 1.014(0.965,1.065) |

Model 1. Unadjusted model

Model 2. Model 1 further adjusted for age, gender, residential type, marital status, economic situation and education

Model 3. Model 2 further adjusted for some chronic diseases, ADL and self-reported health

**Table S2. *HRs* and 95% *CIs* for the mortality risks of healthy lifestyle among the elderly by age in China**

|  | 65- | 75- | 85- | 95- |
| --- | --- | --- | --- | --- |
|  | *HR（95%CI）* | *HR（95%CI）* | *HR（95%CI）* | *HR（95%CI）* |
| Drinking |  |  |  |  |
| Never | 1 | 1 | 1 | 1 |
| Former | 1.396(1.106,1.762) | 1.082(0.932,1.257) | 0.976(0.877,1.087) | 1.115(0.984,1.264) |
| Current | 0.909(0.730,1.132) | 0.864(0.749,0.996) | 0.982(0.891,1.082) | 1.023(0.914,1.145) |
| Smoking |  |  |  |  |
| Never | 1 | 1 | 1 | 1 |
| Former | 1.764(1.399,2.225) | 1.423(1.231,1.645) | 1.214(1.099,1.340) | 1.185(1.047,1.341) |
| Current | 1.469(1.193,1.808) | 1.295(1.132,1.482) | 1.152(1.046,1.269) | 0.980(0.857,1.120) |
| Physical activity |  |  |  |  |
| Current | 1 | 1 | 1 | 1 |
| Former | 1.246(0.947,1.639 | 1.245(1.039,1.491) | 1.351(1.200,1.570) | 1.137(0.996,1.298) |
| Physical inactivity | 0.874(0.733,1.042) | 1.186(1.057,1.331) | 1.171(1.078,1.272) | 1.170(1.058,1.295) |
| Eat fruits |  |  |  |  |
| Yes | 1 | 1 | 1 | 1 |
| Rarely or never | 1.164(0.952,1.424) | 1.168(1.039,1.313) | 1.029(0.954,1.111) | 1.048(0.964,1.138) |
| Eat vegetable |  |  |  |  |
| Yes | 1 | 1 | 1 | 1 |
| Rarely or never | 1.835(0.971,3.468) | 1.147(0.804,1.638) | 1.238(0.991,1545) | 1.190(0.996,1.422) |
| BMI |  |  |  |  |
| Normal weight(18.5-24.9kg/m²) | 1 | 1 | 1 | 1 |
| Underweight  (<18.5kg/m²) | 1.242(1.011,1.526) | 1.134(1.009,1.274) | 1.073(0.998,1.154) | 1.001(0.928,1.080) |
| Overweight (25.0-29.9 kg/m²) | 0.804(0.620,1.042) | 0.916(0.753,1.114) | 1.138(0.976,1.327) | 0.953(0.791,1.147) |
| Obese(≥30.0 kg/m²) | 0.372(0.139,0.998) | 0.819(0.506,1.325） | 0.981(0.687,1.401) | 0.936(0.659,1.330) |
| Sleep duration |  |  |  |  |
| 7-9 hours | 1 | 1 | 1 | 1 |
| <7/>9 hours | 0.958(0.813,1.129) | 1.083(0.977,1.199） | 1.046(0.976,1.327) | 1.127(1.044,1.217) |

**Table S3 *HRs* and 95%*CIs* for the mortality risks of lifestyle among the elderly in China**

|  | Model 1 | Model 2 | Model 3 |
| --- | --- | --- | --- |
|  | *HR（95%CI）* | *HR（95%CI）* | *HR（95%CI）* |
| Risk score |  |  |  |
| 0 | 1 | 1 | 1 |
| 1 | 1.086(0.939,1.255) | 1.080(0.934,1.249) | 1.053(0.911,1.219) |
| 2 | 1.324(1.153,1.520) | 1.143(0.994,1.315) | 1.101(0.958,1.267) |
| 3 | 1.447(1.260,1.662) | 1.230(1.069,1.415) | 1.165(1.012,1.341) |
| 4 | 1.432(1.238,1.656) | 1.248(1.077,1.447) | 1.166(1.005,1.352) |
| 5 | 1.733(1.464,2.052) | 1.470(1.237,1.748) | 1.360(1.144,1.617) |
| 6 | 1.862(1.373,2.525) | 1.500(1.104,2.039) | 1.321(0.791,1.796) |

Model 1. Unadjusted model

Model 2. Model 1 further adjusted for age, gender, residential type, marital status, economic situation and education

Model 3. Model 2 further adjusted for some chronic diseases, ADL and self-reported health

**Table S4 *HRs* and 95%*CIs* for the mortality risks of lifestyle among the elderly by age in China**

|  | 65- | 75- | 85- | 95- |
| --- | --- | --- | --- | --- |
|  | *HR (95%CI)* | *HR (95%CI)* | *HR (95%CI)* | *HR (95%CI)* |
| Risk score |  |  |  |  |
| 0 | 1 | 1 | 1 | 1 |
| 1 | 0.829(0.529,1.298) | 1.004(0.770,1.309) | 1.184(0.956,1.465) | 1.006(0.775,1.307) |
| 2 | 1.247(0.815,1.907) | 1.147(0.893,1.473) | 1.238(1.010,1.519) | 0.999(0.777,1.286) |
| 3 | 1.485(0.970,2.273) | 1.241(0.966,1.594) | 1.283(1.046,1.573) | 1.128(0.877,1.451) |
| 4 | 1.247(0.792,1.966) | 1.428(1.097,1.858) | 1.393(1.128,1.721) | 1.211(0.933,1.572) |
| 5 | 1.325(0.738,2.381) | 1.786(1.313,2.430 | 1.580(1.244,2.008) | 1.315(0.973,1.777) |
| 6 | 0.944(0.128,6.980) | 1.961(1.178,3.264) | 1.600(1.089,2.349) | 1.244(0.639,2.421) |

**References:**

1.Danaei G, Ding EL, Mozaffarian D, Taylor B, Rehm J, Murray CJ, et al. The preventable causes of death in the United States: comparative risk assessment of dietary, lifestyle, and metabolic risk factors. PLoS medicine. 2009;6(4):e1000058.

2.Ford ES, Zhao G, Tsai J, Li C. Low-risk lifestyle behaviors and all-cause mortality: findings from the National Health and Nutrition Examination Survey III Mortality Study. American journal of public health. 2011;101(10):1922-9.

3.McGinnis JM, Foege WH. Actual causes of death in the United States. Jama. 1993;270(18):2207-12.

4.Mokdad AH, Marks JS, Stroup DF, Gerberding JL. Actual causes of death in the United States, 2000. Jama. 2004;291(10):1238-45.

5.Alcohol use and burden for 195 countries and territories, 1990-2016: a systematic analysis for the Global Burden of Disease Study 2016. Lancet (London, England). 2018;392(10152):1015-35.

6.Lee YM, Bae SG, Lee SH, Jacobs DR, Jr., Lee DH. Associations between cigarette smoking and total mortality differ depending on serum concentrations of persistent organic pollutants among the elderly. Journal of Korean medical science. 2013;28(8):1122-8.

7.Foster HME, Celis-Morales CA, Nicholl BI, Petermann-Rocha F, Pell JP, Gill JMR, et al. The effect of socioeconomic deprivation on the association between an extended measurement of unhealthy lifestyle factors and health outcomes: a prospective analysis of the UK Biobank cohort. The Lancet Public health. 2018;3(12):e576-e85.

8.Loef M, Walach H. The combined effects of healthy lifestyle behaviors on all cause mortality: a systematic review and meta-analysis. Preventive medicine. 2012;55(3):163-70.

9.Wu MY, Wang JB, Zhu Y, Lu JM, Li D, Yu ZB, et al. Impact of Individual and Combined Lifestyle Factors on Mortality in China: A Cohort Study. American journal of preventive medicine. 2020;59(3):461-8.

10.Knoops KT, de Groot LC, Kromhout D, Perrin AE, Moreiras-Varela O, Menotti A, et al. Mediterranean diet, lifestyle factors, and 10-year mortality in elderly European men and women: the HALE project. Jama. 2004;292(12):1433-9.

11.Liu Y, Wheaton AG, Chapman DP, Croft JB. Sleep duration and chronic diseases among U.S. adults age 45 years and older: evidence from the 2010 Behavioral Risk Factor Surveillance System. Sleep. 2013;36(10):1421-7.

12.Yin J, Jin X, Shan Z, Li S, Huang H, Li P, et al. Relationship of Sleep Duration With All-Cause Mortality and Cardiovascular Events: A Systematic Review and Dose-Response Meta-Analysis of Prospective Cohort Studies. Journal of the American Heart Association. 2017;6(9).

13.Zhang QL, Zhao LG, Zhang W, Li HL, Gao J, Han LH, et al. Combined Impact of Known Lifestyle Factors on Total and Cause-Specific Mortality among Chinese Men: A Prospective Cohort Study. Scientific reports. 2017;7(1):5293.

14.Obesity: preventing and managing the global epidemic. Report of a WHO consultation. World Health Organization technical report series. 2000;894:i-xii, 1-253.

15.Li Y, Schoufour J, Wang DD, Dhana K, Pan A, Liu X, et al. Healthy lifestyle and life expectancy free of cancer, cardiovascular disease, and type 2 diabetes: prospective cohort study. BMJ (Clinical research ed). 2020;368:l6669.

16.Katz S, Ford AB, Moskowitz RW, Jackson BA, Jaffe MW. STUDIES OF ILLNESS IN THE AGED. THE INDEX OF ADL: A STANDARDIZED MEASURE OF BIOLOGICAL AND PSYCHOSOCIAL FUNCTION. Jama. 1963;185:914-9.

17.Lin YH, Ku PW, Chou P. Lifestyles and Mortality in Taiwan: An 11-Year Follow-up Study. Asia-Pacific journal of public health. 2017;29(4):259-67.

18.Park LG, Dracup K, Whooley MA, McCulloch C, Lai S, Howie-Esquivel J. Sedentary lifestyle associated with mortality in rural patients with heart failure. European journal of cardiovascular nursing : journal of the Working Group on Cardiovascular Nursing of the European Society of Cardiology. 2019;18(4):318-24.

19.Rizzuto D, Fratiglioni L. Lifestyle factors related to mortality and survival: a mini-review. Gerontology. 2014;60(4):327-35.

20.Aune D, Sen A, Prasad M, Norat T, Janszky I, Tonstad S, et al. BMI and all cause mortality: systematic review and non-linear dose-response meta-analysis of 230 cohort studies with 3.74 million deaths among 30.3 million participants. BMJ (Clinical research ed). 2016;353:i2156.

21.Bhaskaran K, Dos-Santos-Silva I, Leon DA, Douglas IJ, Smeeth L. Association of BMI with overall and cause-specific mortality: a population-based cohort study of 3·6 million adults in the UK. The lancet Diabetes & endocrinology. 2018;6(12):944-53.

22.Global BMIMC, Di Angelantonio E, Bhupathiraju Sh N, Wormser D, Gao P, Kaptoge S, et al. Body-mass index and all-cause mortality: individual-participant-data meta-analysis of 239 prospective studies in four continents. Lancet (London, England). 2016;388(10046):776-86.

23.Wändell PE, Carlsson AC, Theobald H. The association between BMI value and long-term mortality. International journal of obesity (2005). 2009;33(5):577-82.

24.Flegal KM, Graubard BI, Williamson DF, Gail MH. Cause-specific excess deaths associated with underweight, overweight, and obesity. Jama. 2007;298(17):2028-37.

25.Kee CC, Sumarni MG, Lim KH, Selvarajah S, Haniff J, Tee GHH, et al. Association of BMI with risk of CVD mortality and all-cause mortality. Public health nutrition. 2017;20(7):1226-34.

26.Tsai AC, Hsiao ML. The association of body mass index (BMI) with all-cause mortality in older Taiwanese: results of a national cohort study. Archives of gerontology and geriatrics. 2012;55(2):217-20.

27.Ng TP, Jin A, Chow KY, Feng L, Nyunt MSZ, Yap KB. Age-dependent relationships between body mass index and mortality: Singapore longitudinal ageing study. PloS one. 2017;12(7):e0180818.

28.He Y, Jiang B, Li LS, Li LS, Sun DL, Wu L, et al. Changes in smoking behavior and subsequent mortality risk during a 35-year follow-up of a cohort in Xi'an, China. American journal of epidemiology. 2014;179(9):1060-70.

29.Reid MC, Boutros NN, O'Connor PG, Cadariu A, Concato J. The health-related effects of alcohol use in older persons: a systematic review. Substance abuse. 2002;23(3):149-64.

30.Rehm J, Roerecke M, Room R. All-Cause Mortality Risks for "Moderate Drinkers": What Are the Implications for Burden-of-Disease Studies and Low Risk-Drinking Guidelines? Journal of studies on alcohol and drugs. 2016;77(2):203-4; discussion 5-7.

31.Ding D, Rogers K, van der Ploeg H, Stamatakis E, Bauman AE. Traditional and Emerging Lifestyle Risk Behaviors and All-Cause Mortality in Middle-Aged and Older Adults: Evidence from a Large Population-Based Australian Cohort. PLoS medicine. 2015;12(12):e1001917.
